# A Study to investigate the role of p27 and Cyclin E immunoexpression as a prognostic factor in early breast carcinoma

**DOI:** 10.1186/1477-7819-9-31

**Published:** 2011-03-16

**Authors:** Komala Pillay, Heather McCleod, Runjan Chetty, Pauline Hall

**Affiliations:** 1 Department of Anatomical Pathology,NHLS, Red Cross Chidren's Hospital/Groote Schuur Hospital, University of Cape Town, South Africa; 2 Biomedical Research Centre, Oxford Radcliffe Hospitals NHS Trust and University of Oxford, John Radcliffe Hospital, UK

## Abstract

**Background:**

Cyclin E and p27 expression is easy to assess in human tissues by standard immunohistochemical techniques. Immunohistochemistry is cost effective, relatively easy to perform and will play more of a role in the future management of cancer. The aim of this study was to investigate the role of p27 and cyclin E immunoexpression as a prognostic factor in early breast carcinoma.

**Methods:**

Cyclin E and p27 immunohistochemistry was performed on sixty six cases of breast carcinoma submitted over a five year period to the Division of Anatomical Pathology, Groote Schuur hospital; Whittaker and Associates; and PathCare. All tumours included in this study were less than 5 cm in diameter (pT1 and pT2 stage) and all the patients had wide local excisions performed. Follow up information was obtained from patient folders in the Department of Radiation Oncology.

**Results:**

There was no significant association of cyclin E and p27 expression with distant metastasis free survival (MFS) for all invasive carcinomas in contrast to grade, lymph node spread and vascular invasion. However, there was a statistically significant direct association of cyclin E with distant metastases in all invasive carcinomas, in the subgroup of infiltrating duct carcinomas (IDC) and in the node negative group when cyclin E was stratified as negative and positive (low/high). In this study of early breast carcinoma, only 9/66 cases showed cyclin E expression. Of these, four patients had distant metastases, one patient had a local recurrence and four patients were alive at last follow-up. Furthermore, cyclin E expression was significantly associated with grade, lymph node spread, oestrogen receptor status and histological type. None of the lobular carcinomas showed cyclin E positivity and only one case of lobular carcinoma presented with distant metastases.

59/66 cases were positive (low/high) for p27 while seven cases were negative, 22 cases showed low expression and 37 cases demonstrated high p27 expression.

p27 was significantly associated with oestrogen receptor status only for all invasive carcinomas and in the IDC group. There was no statistical relationship between p27 and cyclin E, but 50 (76%) tumours with positive p27 expression were negative for cyclin E. There were similar results for the invasive ductal carcinoma subgroup.

**Conclusion:**

This study shows that p27 and cyclin E are not good independent prognostic markers for early breast carcinoma in contrast to grade, lymph node spread and vascular invasion for all invasive carcinomas. However, cyclin E provides some prognostic value as there is a direct statistical association with the development of distant metastases. Many previous studies have correlated overexpression of cyclin E with an aggressive course. The inverse relationship between p27 and cyclin E expression which has been reported in the literature has been highlighted, but this was not statistically significant. Most cases showed positive p27 expression and negative Cyclin E expression. This may be due to the early stage of the disease.

## Background

Cyclin E and p27 expression are easy to assess in human tissues by standard immunohistochemical techniques. Immunohistochemistry is cost effective, relatively easy to perform and will play more of a role in the future management of cancer [1].

The cell cycle is fundamental to all eukaryotic cells and it has been the focus of many studies [2]. An abnormal cell cycle is central to the development of neoplastic conditions. The phases of the cell cycle are G1 (cells prepare their machinery for replication), S phase (duplication of genomic material), G2 (intervening phase), and the M phase (mitosis). Cyclins combine with cyclin-dependent kinases to form heterodimeric molecules, which ensure orderly progression through the different phases of the cell cycle [2]. Two families of cyclin dependent kinase (CDK) inhibitors negatively regulate CDK activities and mediate arrest of the cell cycle following growth inhibitory stimuli. The INK4 (inhibitor of CDK4) family members are p15, p16, p18 and p19 and the kinase inhibitor family (KIP) are p21, p27 and p57 [3].

The G1 to S restriction point is one of the most studied and overexpression of cyclin E shortens the length of G1, accelerating progression of the cell cycle into the S phase [4]. The activation of cyclin E is mediated through its activation of the cyclin dependant kinase 2 protein and is modulated by the presence of cyclin dependent kinase inhibitors such as p27 [5]. It has been shown that accumulation of cyclin E up to a critical level is necessary for initiation of DNA replication [6]. Another study also showed that a high expression of cyclin E promoted cell growth and DNA synthesis and accelerated progression from the G1 phase to S phase [7]. Thus, overexpression of cyclin E or loss of p27 protein expression may result in tumour development and/or progression.

Both p27 and cyclin E expression have been examined in many malignancies, including breast carcinomas [8-13].

*Cyclin E *is located on chromosome 19q12-13 and the two E-type cyclins, cyclin E1 and E2, are collectively referred to as cyclin E [13,14]. They are closely related and often co-expressed. During the G1 phase, CDK2 is activated through binding cyclin E and, via phosphorylation of target proteins, facilitates the progession into the S-phase [15]. Cyclin E2 shares 47% overall similarity to cyclin E1 (cyclin E) and also associates with CDK2. Recently, several splice variants of cyclin E1 which are not present in normal cells have also been discovered which seem to stimulate the cells to progress through the cell cycle more efficiently than the full-length cyclin E through a mechanism that is not yet completely understood [14].

A strong correlation has been demonstrated between increased cyclin E expression and human breast carcinoma. Increased expression of cyclin E has been reported in approximately 40% of primary oestrogen receptor negative breast carcinoma [14].

p27 is an important cell cycle regulatory protein that belongs to the Cip/Kip family [16]. It plays an important role in the progression from the G1 to S phase of the cell cycle by inhibiting the CDKs and may act as a tumour suppressor. p27 is a potent inhibitor of cyclin E/CDK 2 and cyclin A/CDK2 and its expression is highest in quiescent cells and decreases upon re-entry into the cell-cycle [16]. Increase in p27 is associated with cell growth arrest, cell differentiation or an increase in apoptotic activities whereas decreased p27 expression is related to increased proliferation and tumorigenesis [17]. The p27 gene maps to position 14q32 on the human genome [18]. p27 mutations are a rare event in breast cancer [19]. Although a minority of breast carcinomas may show mutations, most of the p27 abnormalities occur at a post-transcriptional level [20].

The aim of this study was to investigate the role of p27 and cyclin E immunoexpression as a prognostic factor in early breast carcinoma.

## Methods

A database of all wide local excisions for breast carcinoma from 1995 to 1999 was used from the Department of Radiation Oncology which comprised 88 cases. Of these, paraffin blocks were available for 66 cases.

The blocks for these cases were retrieved from the Department of Anatomical Pathology, Groote Schuur hospital (GSH), Whittaker and Associates, and PathCare (ex Dietrich, Street and Partners). All tumours included in this study were less than 5 cm (pT1 and pT2 stage) and all the patients had wide local excisions performed. The slides and blocks were retrieved from the archives of the Anatomical Pathology Department, GSH and the two private practices. Follow up information was obtained from patient folders in the Department of Radiation Oncology. Staging was done using the American Joint Committee on Cancer (AJCC) Staging System for breast cancer (1992 revision) based on a combination of clinical information (such as bone scans), histopathology and cytology.

Immunohistochemical studies were performed on 66 formalin-fixed, paraffin embedded surgical specimens. Representative sections, cut between two and four microns, were mounted onto positively charged slides to prevent lifting of sections during heat induced epitope retrieval (HIER). Epitope retrieval involved bringing the specific buffer to boiling point in a pressure cooker, immersing dewaxed sections in boiling buffer and sealing the pressure cooker. When maximum pressure was reached, it was maintained for two minutes. Thereafter, the pressure cooker was immediately kept under running tap water to break the vacuum and slides were removed into running tap water till the next step.

Endogenous peroxidase activity was prevented by treating slides with 1% aqueous hydrogen peroxide (H_2_O_2_) for 15 minutes. Staining took place at room temperature and phosphate buffered saline (PBS) with 0.05% Tween used for all rinse steps. Primary antibodies, p27 and cyclin E, were incubated for 60 mins. See Table 1 for details of primary antibodies. Goat anti-mouse immunoglobulin/peroxidase (Envision) was applied for 40 minutes while the biotinylated goat anti-mouse immunoglobulin (LSAB kit) was applied for 20 mins. The colour was developed using DAB (3_'_3'- diaminobenzidine) liquid substrate for 5-10 minutes.

**Table 1:** Antibodies

	Source	Clone	Dilution	Retrieval Buffer	Detection System
**p27**	Novocastra	13A3	1 : 40	10 mM citrate buffer pH6	Envision
	
					Dakocytomation K4001

**cyclin E**	Novocastra	1B4	1 : 50	1 mM EDTA buffer pH8	LSAB
	
					Dakocytomation K0672

Details of p27 and cyclin E antibodies.

The slides were counterstained with haematoxylin, dehydrated, cleared and mounted in Entellan.

Placenta and squamous cell carcinoma of the oesophagus were used as cyclin E and p27 positive controls respectively. In addition, there was p27 expression in benign breast elements, ductal carcinoma in-situ and quiescent lymphocytes. For negative controls, the primary antibody was omitted.

The immunostained sections were graded as negative, low and high, depending on the percentage of nuclei that showed positive staining according to the following scheme. 0 (negative) = positive staining in less than 5% of nuclei); low staining = moderate to strong positive staining in 5% to 50% of nuclei; high staining = moderate to strong positive staining in more than 50% of nuclei.

Statistical analysis was performed using the chi-squared test and the significant p- value is </= 0.05. Distant metastasis free survival (MFS) was assessed using the Proportional hazard (Cox) regression model.

## Results

### Descriptive analysis

The age of the patients ranged from 23 to 82 years. There were 21 white, 41 mixed- race and 4 black patients

The average follow-up period was 53 months with a range of two to 98 months and 52 patients had a follow up of more than 36 months. There were 59 infiltrating ductal carcinomas (not otherwise specified and variants) and 7 infiltrating lobular carcinomas. The infiltrating ductal carcinoma group included cases not otherwise specified (50), mucinous (3), tubular (3), medullary (1), metaplastic (1) and microinvasive (1).

Ten patients had lymph node spread out of the 52 patients with axillary dissections (blocks for immunohistochemistry were only available in nine cases). Thirty four patients were stage 1 and 32 were stage 2 at the time of presentation. Distant metastases developed in 13 cases between 14 and 60 months after presentation. Seven patients died, of whom six had distant metastases; one patient died of dehydration following chemotherapy.

p27 expression was expressed in benign breast tissue and resting lymphocytes which served as good internal control

**Figure 1:**
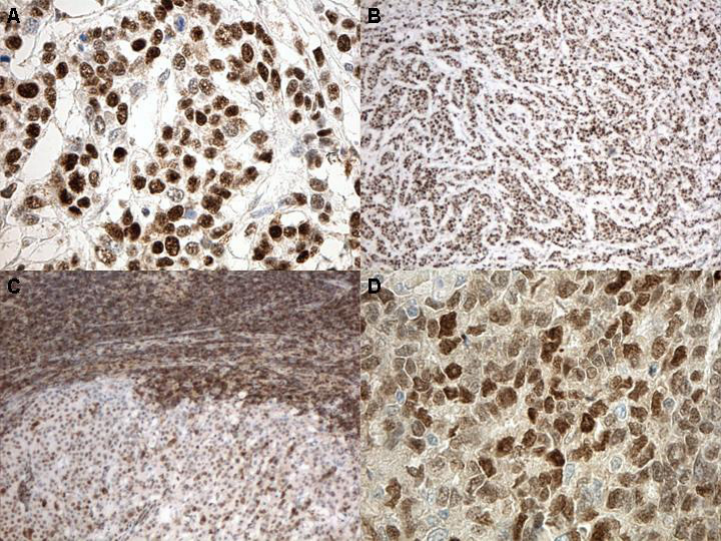
**p27 and cyclin E immunoexpression**. A: Positive p27 expression in an infiltrating duct carcinoma, NOS, B: Positive p27 expression in an infiltrating lobular carcinoma, NOS (100×), C: Low p27 expression in a breast carcinoma metastatic to a lymph node. The lymphocytes are also strongly positive. (40×), D: Positive cyclin E expression in infiltrating duct carcinoma, NOS (100×).

Fifty nine out of 66 cases were positive for p27 in the primary carcinoma: Low:22, high:37, negative:7. p27 expression in nine lymph node metastases was as follows: Low:4, high:5. Nine out of 66 cases were positive for cyclinE: Low:4, high:5; negative:57 (Figure 1)

The expression of cyclin E in the 9 lymph node metastases is: Low:2, negative:7.

### Statistical analysis

The average age of patients with distant metastases (44 years) was 10 years younger than patients without distant metastases (54 years) [p = 0.02] in the IDC group. For all invasive carcinomas, the average age of patients with distant metastases was 46 years compared to 53 years for patients without distant metastases.

There was no difference in cyclin E and p27 expression among the different race groups for all invasive carcinomas (p = 0.38 and p = 0.54, respectively) and in the IDC subgroup (p = 0.47 and p = 0.89 respectively)

Size was statistically associated with the presence of disease (distant metastases, local recurrence and death) [p = 0.04]. 34/48 (71%) of patients that were alive without disease had pT1 lesions whereas only 44% of patients with disease had pT1 lesions. However, there was no significant relationship of size with p27 and cyclin E expression (see later).

**Figure 2:**
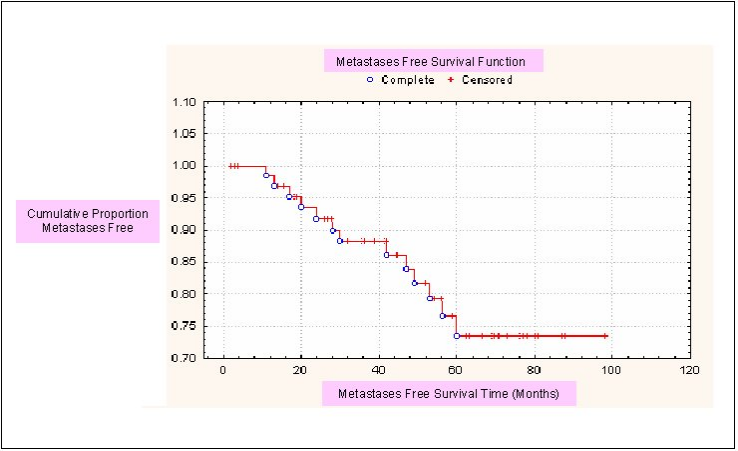
**Distant metastases**. Graph showing the number of distant metastases over a period of time (distant metastases free survival). Complete (o) = distant metastases Censored (+) = no distant metastases.

Thirteen of the 66 patients presented with distant metastases, all within five years of the date of first presentation (Figure 2).

There was no difference in MFS (distant metastases free survival) amongst the different races (p = 0.41)

A univariate analysis showed a significant relation between MFS and age, grade, lymph node spread and vascular invasion. The results were similar for IDC (infiltrating ductal carcinomas). Grade, lymph node spread and vascular invasion were still significant in a stepwise multivariate analysis for all invasive carcinomas and the subgroup of IDC (Table 2).

**Table 2:** Prognostic factors

	p-values for all invasive carcinomas	p-values for infiltrating duct carcinomas only
**Univariate analysis**		

Age	*0.047*	*0.02*

Grade	*0.007*	*0.03*

Lymph node spread	*0.0008*	*0.004*

Vascular invasion	*0.001*	*0.004*

Oestrogen receptor status	0.47	0.29

Adequacy of resection	0.64	0.18

p27 expression in the primary tumour	0.37	0.26

Cyclin E expression in the primary tumour	0.31	0.35

**Multivariate analysis**		

Grade	*0.05*	*0.02*

Lymph node spread	*0.04*	*0.02*

Vascular invasion	*0.04*	*0.04*

p-values for prognostic factors.

Further univariate analyses revealed no significant association of MFS with oestrogen receptor status, adequacy of resection, p27 expression and cyclin E expression in the primary tumour for all invasive carcinomas. The subgroup of IDC showed similar features (Table 2).

There was a significant direct relationship of Cyclin E expression with distant metastases for all invasive carcinomas and the IDC group when cyclin E was stratified as negative and positive (low/high) (Table 3). In the IDC group, four out of the eight patients with distant metastases had positive cyclin E. Of note, of the five patients with positive Cyclin E and no metastases, one patient developed local recurrence and four patients were alive at last follow up.

**Table 3:** p27 and cyclin E statistical associations

	p27		Cyclin E	
	**All invasive carcinomas**	**Infiltrating duct carcinomas only**	**All invasive carcinomas**	**Infiltrating duct carcinomas only**

Distant metastases	0.85	0.54	*0.04*	*0.02*

Grade	0.30	0.08	*0.01*	*0.009*

Lymph node spread	0.66	0.72	*0.05*	0.29

Oestrogen receptor status	0.056 *(0.04)*	*0.017*	*0.002*	*0.002*

Histological type	0.23		*0.01*	

Tumour size	0.08	0.22	0.80	0.42

p-values for the association of p27 and cyclin E expression with other prognostic factors

However, there was no significant relationship of p27 expression with distant metastases in both groups (Table 3). There was also no relationship between p27 and most of the other prognostic markers: Lymph node spread, grade, tumour size and histological type. The association with oestrogen receptor status showed near significance (Table 3). However, when p27 was stratified as negative/low (in one group) and high, there was a significant relationship with ER status (p = 0.04).

There was no relationship between p27 and most of the other prognostic markers in the IDC subgroup (Table 3).

In this subgroup of IDC, the association with oestrogen receptor status showed significance (p = 0.017). Seventy four percent (25/34) of cases with high p27 expression were positive for oestrogen receptors.

Cyclin E expression in the primary tumour showed significant association with grade, lymph node spread, oestrogen receptor status and histological type.

Eight out of nine (89%) Grade 3 tumours had positive (low/high) cyclin E expression whereas none of the Grade 1 tumours displayed positive cyclin E expression.

100% (24/24) of grade 1 tumours, 86% (36/42) of node negative cases and 98% (40/41) of tumours with positive oestrogen receptors had negative cyclin E expression. In contrast, 95% (39/41) of oestrogen receptor positive cases showed p27 positivity.

All of the lobular carcinomas were negative for cyclin E. In other words, all the Cyclin E positive cases were infiltrating duct carcinomas. There was no significant correlation between cyclin E and tumour size (near significance).

In the subgroup of IDC, there was significant association of cyclin E with grade and oestrogen receptor status only. There was no significant correlation between cyclin E and lymph node spread and tumour size.

There was no significant correlation between p27 expression and cyclin E expression p = 0.22. However, 50 (76%) tumours with positive p27 expression were negative for cyclin E. There were similar results with the IDC subgroup.

### Node negative group

Using the Proportional hazard (Cox) regression model in the node negative group, there was still no association of p27 and cyclin E with MFS for all invasive carcinomas (p = 0.26 and p = 0.46 respectively) and IDC group (p = 0.17 and p = 0.56 respectively).

In the node negative group for all invasive carcinomas, Cyclin E expression had a statistically significant relationship with the development of metastatic disease (p = 0.04). 89% (32/36) of patients with no metastases and 67% (4/6) of patients with metastases showed negative cyclin E expression. This group showed no relationship between p27 expression and the development of distant metastatic tumour.

In the IDC group, cyclin E was statistically associated with distant metastases (p = 0.05) whereas p27 was still not associated with distant metastases (p = 0.41). 88% (29/33) of patients with negative cyclin E expression did not develop distant metastases whereas only 67% (4/6) of patients with negative cyclin E developed distant metastases.

## Discussion

p27 and cyclin E have been examined in many malignancies, including breast carcinomas [8-13,21-23]. Although it is generally felt that cyclin E overexpression and decreased p27 expression is associated with an adverse prognosis the results of studies vary and it seems that more research is required before p27 and cyclin E are accepted or rejected as prognostic markers in breast carcinoma [1,13].

This study analysed 66 cases of small breast carcinomas, less than 5 cm (pT1 and pT2 stage) where the treatment was wide local excision with or without axillary lymph node dissection. Studies of early breast cancer are important to help discover markers that may have a prognostic impact and thus have an influence on the choice of adjuvant therapy.

In this study, 29/66 (44%) cases showed low or negative p27 expression. This prevalence of reduced p27 immunoreactivity is consistent with that reported in the previous studies, ranging from 31-69% [24]. Spataro et al. feel that down-regulation of p27 is likely to be an early event in breast cancer as they detected it with the same prevalence in small lymph node negative tumours with limited invasion and in larger lymph node positive groups [24].

In this study, there was a significant association of age, grade, lymph node spread and vascular invasion with distant metastases free survival (MFS) in all invasive carcinomas and the subgroup of IDC in an univariate analysis. However, there was no significant association of oestrogen receptor status, adequacy of resection, p27 and cyclin E expression in the primary tumour with MFS. This is similar to some studies that did not find a relationship of p27 and cyclin E expression with prognosis in breast carcinomas [8,12,25].

In this study, only 13.6% of breast carcinomas showed cyclin E positivity. This may be due to the small size of the tumours. In the studies by Donnellan et al. and Kim et al. cyclin E was positive in 46% and 41% of patients respectively [8,10]. However, there was no restriction in size of tumours in these studies.

Barbareschi et al. analysed p27 expression in 512 breast carcinoma cases with a follow up of 10 years and concluded that no prognostic value was seen in the subgroup of small tumours nor in the group of young patients and the results of the node positive and node negative patients were not statistically different [26].

However initial papers on p27 expression in breast carcinoma by Tan et al. (studied T1a,b lesions only), Catzavelos et al. and Porter et al. (22-44 year old patients only) showed a striking association between loss of p27 and poor prognosis [21-23]. Interestingly, Tan et al. looked at very small carcinomas up to one centimetre in greatest dimension in a large cohort of 202 cases [21]. In their study, oestrogen and progesterone receptor status and Her-2/neu were not significantly associated with survival by univariate analysis. But the level of p27 expression was associated significantly with survival with a median survival of 174 months in patients whose tumours displayed high p27 and 139 months in tumours with low p27 (p = 0.0042). Significance was maintained when node positive patients were excluded implying p27 expression yields prognostic information in node-negative patients. Thus, patients with small invasive carcinomas who may benefit from adjuvant therapy can be identified.

Porter et al. characterised the expression of cyclin E and p27 in breast carcinomas from 278 patients [23]. In this study, positive lymph nodes, large tumour size, intermediate and high histologic grade, presence of Her-2/neu, high levels of cyclin E and low or absent p27 were associated with increased risk of death in univariate models. However, only lymph node status, presence of Her-2/neu, high cyclin E levels and low p27 levels were associated with decreased survival after adjusting for all factors. High p27 and low cyclin E was associated with the best survival whereas the opposite pattern (low p27 and cyclin E) was associated with the highest mortality. Of note, the greatest difference in survival was found between patients having almost no p27 and patients having the highest levels.

Another study of exclusively small tumours is by Blegen et al. who performed a genetic study on microdissected tissue from 33 primary breast carcinomas, stage T1b and T1c, looking at DNA content, chromosomal gains and losses, p27 and cyclin A among other factors [27]. In this study, high level chromosomal copy number increases (amplifications) correlated with elevated cyclin A and reduced p27 expression.

Gillet et al. showed that p27 is prognostically relevant at univariate analysis, but not at multivariate analysis [11]. This study also demonstrated that the value of p27 is strongly dependent upon its association with grade.

The study by Kim et al. suggested that cyclin E overexpression in primary breast carcinoma could independently predict distant relapse as the first failure after curative breast surgery[10]. In their study of 128 cases of breast carcinoma of all sizes, distant relapse could be predicted by lymph node spread, high cyclin E expression and the younger age (< 35 years) of patients. When specific types of metastases were analysed, high cyclin E predicted the risk of distant metastases with borderline significance on multivariate analysis whereas both overexpressed cyclin E and the younger age of patients were independent risk factors for visceral relapse. Of note, in the current study, patients with distant metastases were about 10 years younger than patients without distant metastases (p = 0.02).

In this study, there was a significant direct association of cyclin E with distant metastases in all invasive carcinomas, in the IDC group only and in the node negative group when cyclin E was stratified as negative and positive (low/high). But p27 expression was not significantly associated with distant metastases. In one study, all node negative patients with high levels of cyclin E (12 out of 114) died of breast carcinoma [28]. In this study, 9/66 cases showed cyclin E expression. Of these, four patients had distant metastases, one patient had local recurrence and four patients were alive at last follow-up.

In this study, cyclin E expression was significantly associated with grade, lymph node spread, oestrogen receptor status and histological subtype for all invasive carcinomas. These are factors that are also easily assessed by morphological assessment. Donnellan et al. concluded that cyclin E appeared to contribute to prognosis in breast ductal carcinomas primarily through its contribution to proliferation which is already assessed by tumour grading [8]. However, their cohort of patients had many poor risk factors and they suggested that a greater number of cases were required to ascertain whether cyclin E immunostaining improves assessment of prognosis in node negative patients.

p27, on the other hand, showed no significant association with lymph node spread, grade, tumour size and histological type in all invasive carcinomas and the subgroup of IDC. However, p27 was significantly associated with oestrogen receptor status in both groups. Almost 75% of cases with high p27 expression in both groups were also positive for oestrogen receptors.

A study by Reed et al. on a series of 77 node negative patients showed an association of p27 expression with low tumour grade and positive oestrogen receptor status [12]. The authors also report a tendency towards better survival for tumours with more than 25% of p27 positive tumour cells, but this result did not reach statistical significance.

Barbareschi et al. have also shown that low p27 expression is associated with high grade and oestrogen receptor negativity [26]. It has also been suggested that low p27 is a strong and independent marker of poor clinical outcome.

In this series, there was no difference among the different race groups with regards to MFS. There was also no difference in cyclin E and p27 expression among the different races.

In a study by Porter et al. it was shown that there were racial differences in breast carcinoma. When disease stage and age at diagnosis were adjusted for, it was shown that African American (AA) women have increased odds of having features associated with a poor prognosis, including overexpression of cyclin E and cyclin D1 [29]. Joe et al. studied the expression levels of cyclin D1, p53, p27, and p21 and correlated their expression with oestrogen receptor status in 200 breast cancer cases obtained from AA and Caucasian patients who were matched on age, stage, oestrogen receptor status, and year of diagnosis [30]. They found that cyclin D1, p53, p27, and p21 expression rates were similar in matched cases of AA and Caucasian breast cancer (p values > 0.05). However, cyclin D1 overexpression was significantly associated with oestrogen receptor status in only the Caucasian (p = 0.0005), and not the AA cases (p = 0.07) which suggested a biological difference, which may contribute to the more aggressive phenotype of African American breast cancer.

In the current study, there was a significant association between histological subtype and cyclin E expression. All cases of lobular carcinoma were negative for cyclin E. In other words, all cyclin E positive cases were infiltrating duct carcinomas.

The results of the study conducted by Sasano et al. (21 invasive duct carcinomas and 19 invasive lobular carcinomas) showed that there was no significant difference in the means of the labelling indexes of Ki67, cyclin D1, cyclin E, cdk2, cdk4, oestrogen receptor and progesterone receptor status in invasive ductal and lobular carcinomas [9]. But the labelling index of cyclin D1 correlated with the pathological stage of the disease in invasive lobular carcinomas but not in invasive ductal carcinomas.

Another study evaluated the bio-molecular differences between ductal and lobular carcinomas in 190 in ductal and 67 lobular carcinomas [31]. Of note, there was no significant difference between lobular carcinomas and ductal carcinomas regarding the expression of CDK inhibitors, including p27. In infiltrating lobular carcinoma, despite the higher oestrogen receptor positivity percentage compared to IDC, oestrogen receptor status was related only to p27. However, regardless of histological type, there was a statistically significant direct relationship between progesterone receptor status and p27 expression.

Orlando et al. studied the expression of various proteins in typical medullary, 'atypical' medullary and ductal breast carcinoma with similar high proliferation [32]. There was no difference in expression of HER-2/neu, p21, p27, p53 and the number of apoptotic cells in the different types. Also, in their series, patients with a previous medullary carcinoma were not free from the risk of developing a subsequent ductal carcinoma and they felt that defining atypical medullary carcinoma as a distinct entity from ductal carcinoma was not justified.

The inverse relationship of cyclin E and p27 has been highlighted in the literature [13]. However, in this study the association of p27 and cyclin E in all invasive carcinomas and in the subgroup of IDC did not show statistical significance. However, 76% of all tumours with positive p27 expression were negative for cyclin E in all invasive carcinomas and in the subgroup of IDC. This may be due to the early stage of the disease.

The major limitation of this study is the small size of the study group. Further studies with larger numbers of small breast carcinomas are required to establish the role of cyclin E and p27 in early breast carcinomas. It has also been suggested that the failure of some studies to find a prognostic value for p27 might reflect differences in tumour fixation and the prolonged storage time of archival tumour blocks utilized in several studies [14]. Therefore large prospective trials with a uniform methodology for tumour processing, staining and scoring are probably required to definitely establish the prognostic value of p27 and cyclin E in breast carcinoma. A major problem that is encountered in comparing various studies is that there are different definitions for p27 positivity, or different cut-off values are used when scoring is based on the percentage of immunoreactive cells. From this study, it seems that p27 may be scored as negative/low and high i.e. less or more than 50% of positive nuclei. Cyclin E may be classified as negative (less than 5% of positive nuclei) or positive (5-100%) and this, further subdivided as low (5-50%) and high (more then 50%).

## Conclusion

This study shows that p27 and cyclin E are not good independent prognostic markers for early breast carcinoma in contrast to grade, lymph node spread and vascular invasion for all invasive carcinomas. However, cyclin E provides some prognostic value as there is a direct statistical association with the development of distant metastases in all invasive carcinomas, the subgroup of invasive ductal carcinomas and in the node negative group when cyclin E was stratified as negative and positive (low/high). Many previous studies have correlated overexpression of cyclin E with an aggressive course. In this study of early breast carcinoma, 9/66 cases showed cyclin E expression. Of these, four patients had distant metastases, one patient had local recurrence and four patients were alive at last follow-up. Cyclin E was significantly associated with grade, lymph node spread, oestrogen receptor status and histological subtype for all invasive carcinomas. None of the lobular carcinomas showed cyclin E positivity and only one case of lobular carcinoma presented with distant metastases.

59/66 cases were positive (low/high) for p27 while seven cases were negative, 22 cases showed low expression and 37 cases demonstrated high p27 expression. Of note, there was a statistically significant relationship of p27 expression with oestrogen receptor status in all invasive carcinomas and the IDC group as reported in the literature.

There was no difference in cyclin E and p27 expression and distant metastases free survival (MFS) among the different race groups.

The inverse relationship between p27 and cyclin E expression which has been reported in the literature has been highlighted, but this was not statistically significant. Most cases showed positive p27 expression and negative cyclin E expression. This may be due to the early stage of the disease.

## Abbreviations

(MFS): metastasis free survival; (IDC): invasive/infiltrating duct carcinomas; (CDK): cyclin dependent kinase; INK4: (inhibitor of CDK4); (KIP): kinase inhibitor family; (HIER): heat induced epitope retrieval; (PBS): phosphate buffered saline; (LSAB): labeled streptavidin biotin; (DAB): 3_'_3'- diaminobenzidine;

## Competing interests

The authors declare that they have no competing interests.

## Authors' contributions

KP and RC conceived the study. HM optimised the antibodies and performed the stains. KP collected the cases and clinical information, interpreted the stains and results, performed the literature review and wrote the manuscript. RC and PH supervised the study and proof-read the manuscript. All authors read and approved the final manuscript.
